# Sleep Patterns, Social Media Usage, and Dietary Habits during COVID-19 Lockdown in Mexico: A Cross-Sectional Study

**DOI:** 10.3390/bs14100906

**Published:** 2024-10-08

**Authors:** María Elena Acosta Enríquez, Danila Azzolina, Anairotciv De la Cruz Alvarez, Vidalma del Rosario Bezáres Sarmiento, Dario Gregori, Giulia Lorenzoni

**Affiliations:** 1 Facultad de Ciencias de la Salud, Universidad de Montemorelos, Montemorelos 67515, Nuevo León, Mexico; elenamaria@um.edu.mx (M.E.A.E.); anairotciv@hotmail.com (A.D.l.C.A.); 2 Department of Environmental and Preventive Science, University of Ferrara, 44121 Ferrara, Italy; danila.azzolina@unife.it; 3 Facultad de Ciencias de la Nutrición y Alimentos, Universidad de Ciencias y Artes de Chiapas, Tuxtla Gutiérrez 29039, Chiapas, Mexico; vidalma.bezares@unicach.mx; 4 Unit of Biostatistics, Epidemiology and Public Health, Department of Cardiac, Thoracic, Vascular Sciences and Public Health, University of Padova, 35131 Padova, Italy; giulia.lorenzoni@unipd.it

**Keywords:** COVID-19 lockdown, Mexico, dietary habits, sleep quality

## Abstract

During the COVID-19 lockdown, a high prevalence of disruption in lifestyle habits was reported, especially concerning sleep habits. The present study aimed to explore the relationship between lifestyles and the COVID-19 lockdown on subjects living in Mexico. A cross-sectional survey was conducted. An online questionnaire was administered to survey participants. A clustering procedure was performed to identify groups of survey respondents with similar characteristics. There were 155 survey respondents. Most of them were women (83%) of young age. The clustering identified two distinct groups of subjects, i.e., Cluster 1 and 2. The subjects in Cluster 2 were younger, more likely to use social media, and spent much more time watching TV/surfing the internet than subjects in Cluster 1. Furthermore, the prevalence of problems with falling and staying asleep during the lockdown was higher in Cluster 2 than in Cluster 1 (14% in Cluster 2 vs. 4% in Cluster 1) even though nobody from Cluster 2 had sleep problems before the lockdown. The study showed that younger respondents were those more affected by lockdown measures.

## 1. Introduction

The COVID-19 pandemic has severely burdened the healthcare resources [1] of countries worldwide. Fighting the epidemic was even more difficult for low- and middle-income countries like the Central and South America. Such countries were already facing a political and economic transition before the virus spread [2]. Furthermore, epidemiological surveillance in such countries was not as well established as in high-income countries [3]. For these reasons, it was hypothesized that the number of COVID-19 cases and deaths was underestimated in Central and Southern American regions [4]. 

Worldwide, the introduction of containment interventions, from partial to full lockdown, was effective in preventing the spread of the virus [5]. However, the literature indicated that such measures have impacted every aspect of people’s lives, negatively affecting their physical health [6], profoundly affecting lifestyle habits [7] and psychological health [8], requiring specific support programs for the community. 

Systematic reviews in the field showed that the lockdown was associated with an impairment in dietary habits, characterized by a documented decreased consumption of fresh fruits and vegetables in favor of calorically dense, but nutrient-poor, food [9,10]. Also, dietary patterns underwent relevant changes, with an increasing frequency of snacking [10]. In the short term, these changes resulted in weight gain [11], with potential long-term detrimental consequences for subjects’ health. The decreased consumption of fresh food was partially explained by the difficulties in purchasing such foods due to food shortages experienced at the very beginning of the lockdown. However, the increased consumption of empty calories and the increased snacking frequency were interpreted as emotional eating, i.e., the attitude of using food to face stress and anxiety. Such a concept highlights the strict relationship between eating habits and emotional well-being, a phenomenon that has been documented during the lockdown [12]. 

Together with eating habits, emotional well-being was found to undergo a substantial disruption during the COVID-19 lockdown. A higher prevalence of anxiety and major depressive disorders was documented worldwide compared to the pre-lockdown period [13]. The prevalence of such conditions was found to be higher among those countries most severely affected by the pandemic [13].

Not least, changes in lifestyle habits have also been reported. A reduced frequency of physical activity in favor of adopting sedentary behaviors was shown during the lockdown [14,15]. In addition, changes in digital habits, particularly increased use of social media, were reported during the lockdown [16], with a significant increase in screen time documented globally. Social media platforms have become a primary tool for maintaining social connections but also a source of anxiety [17]. Furthermore, this increased use, especially in the evening, has been suggested to be associated with alterations in sleep patterns in pre-pandemic literature [18], such as delayed onset of sleep and decreased sleep quality. 

Alterations in sleep patterns were widely documented in the literature during the lockdown [19]: several sleep problems emerged during the COVID-19 lockdown, including insomnia, difficulty falling asleep, frequent awakenings, and nonrestorative sleep [20]. These disorders were particularly prevalent among populations with pre-existing mental health conditions, healthcare workers, and college students compared to the general population [21]. Again, such problems were found to be associated with psychological distress [19].

The link between lifestyle habits, including eating patterns, sleep quality, and emotional health, is complex and difficult to disentangle. It has been hypothesized that poor sleep would result in emotional dysregulation, leading to poor lifestyle habits, i.e., poor eating patterns and physical activity [22]. The long-term consequences of such changes would be detrimental. For this reason, researchers claimed the need to develop community support programs, especially for the most vulnerable. To do so, the characteristics of subjects more prone to the adverse effects of the COVID-19 lockdown were investigated. Female gender, young age, and low socioeconomic status seem to be risk factors for worse outcomes [13]. 

The present study aimed to explore the relationship of the COVID-19 lockdown with the emotional well-being and lifestyle habits of people in Mexico, particularly focusing on alterations in sleep patterns and social media use during the lockdown. Data for Mexico are still relatively sparse compared to other countries. Recent studies have shown that in Mexico, consistently with more widely studied countries, the lockdown led to a significant increase in social media use and screen time, which was associated with disturbed sleep patterns and higher levels of insomnia [23], together with disruption of eating patterns, especially in low-income subjects [24]. Understanding these impacts is crucial, as they may have long-term consequences for the well-being of young people, particularly considering the specificities of Mexico in facing the pandemic. The lockdown was declared from April to the end of May 2020, but, despite the measures, it was one of the countries with the highest number of COVID-19 cases and deaths [25]. Furthermore, differently from other countries where death cases were most common among the elderly, deaths in Mexico were most often associated with noncommunicable diseases, such as diabetes and overweight/obesity [25], where the prevalence of these diseases is among the highest in the world. 

## 2. Materials and Methods

The study was cross-sectional and collected data through an online survey. A snowball sampling technique was adopted, a sampling strategy widely used during the pandemic period by surveys exploring lifestyle habits and emotional well-being [26,27,28,29]. This method of administration results in a statistical sample with uncontrollable population parameters, unlike probability sampling. However, it proved effective in achieving the research objectives, as it allowed for the dissemination of the survey despite the restrictions. 

Study subjects must be over 18 years of age, living in Mexico during the lockdown, and able to read and understand the Spanish language. The link to the survey was available on the Google online survey platform. It was distributed using messenger apps, i.e., WhatsApp. Messenger apps are widely used in the country; mobile phone users using WhatsApp have been estimated to be more than 80% in the age class 18–34, with only a slight decline in users proportion after age 35 [30]. The data collection took place between 19 April and 2 May 2020. 

The questionnaire was developed explicitly for the study (“Lifestyle in Mexican Families during COVID-19”), Supplementary Material. It was made up of 4 sections. The first part asked survey respondents about socio-demographic characteristics; the second assessed lifestyle habits before and after the lockdown. The third and fourth sections asked participants about social/family relationships during the lockdown and emotional well-being. The development of the questionnaire was guided by a multidisciplinary team, including experts from psychology, public health, and social sciences, who ensured that the questions were relevant and in line with the study’s objectives. Based on previous literature, items were formulated to capture critical aspects of changes in lifestyle, emotional well-being, and social relationships during the lockdown. After the development stage, the questionnaire underwent a pilot testing procedure on a group of twenty subjects selected to be representative of the survey’s target population. 

### Statistical Methods

Data were reported as percentages and absolute numbers. Pearson Chi-square and Fisher exact tests were performed to assess the association between categorical variables.

Clustering is an exploratory technique frequently used in the biomedical sciences. It allows for identifying groups of observations with similar characteristics without an already known grouping criterion. A factorial hierarchical clustering procedure was employed to identify respondents with similar characteristics. 

Before performing the clustering analysis, a multiple correspondence analysis (MCA) procedure was performed. The MCA was used as a data pre-processing step to synthesize data into latent dimensions [31] to be analyzed using the clustering procedure [32]. The MCA worked by transforming the original categorical variables into a few continuous variables, i.e., the latent dimensions, which were subsequently used as input for the clustering procedure.

The hierarchical factorial clustering analysis was subsequently conducted on these latent dimensions identified through the MCA. This method allowed for the hierarchical organization of the data, which were represented in a cluster dendrogram, a tree diagram illustrating the arrangement of clusters produced by the analysis. In addition, cluster membership was represented in a factor map.

These methodological steps ensured the reduction of the complexity of the categorical data and the improvement of the interpretability of the clusters identified in the study.

A significance level of 0.05 was considered for the analyses. Computations were performed with the R 3.6.2 system and the Factominer [33] and Factoextra packages [34].

## 3. Results

There were 155 survey respondents (Table S1, Supplementary Material). The female gender was the most prevalent (83%), and the age group most represented was that of subjects between 21 and 25 years of age (39%). Only 5% of the respondents lived alone. Concerning lifestyle habits during the lockdown, a high proportion of subjects declared watching TV more than 2 h per day (60%) and using social media daily (63%). 

### 3.1. Clustering Results

**Figure 1:**
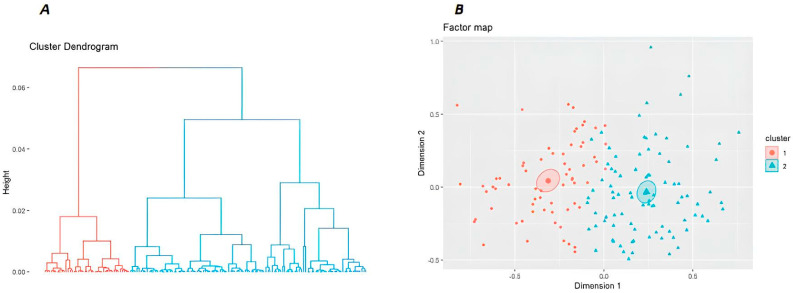
This Figure reports the Cluster Dendogram (**A**) and the Factor Map (**B**) presenting the results of the cluster analysis. In (**A**), the height axis displays the distance (Euclidean) between observations and/or clusters. The horizontal bars indicate the point at which two clusters/observations are merged. The Ward clusters method has been considered with a Manhattan metric for distances. In (**B**), individual loadings are represented according to latent dimensions (Factors). The individual cluster membership is identified according to colors.

Based on the initial sample of 155 survey respondents, the clustering procedure identified two well-characterized groups of 67 and 88 subjects, Cluster 1 and Cluster 2, respectively. Figure 1 provides a graphical representation of the clustering procedure, including the cluster dendogram (A) and the factor map (B). 

Table 1 presents subjects’ characteristics according to the cluster to which they belong.

### 3.2. Clusters’ Demographics

The two clusters differed significantly in age distribution. Half of the subjects (53%) in Cluster 2 were aged 21–25, while the most represented age class of Cluster 1 was 51–60 (*p*-value for age class distribution <0.001). No differences were detected in gender distribution. Concerning the geographical area, a significantly higher proportion of subjects in Cluster 2 compared to Cluster 1 lived in Chiapas (66% vs. 9%, *p*-value < 0.001). In comparison, two-thirds of the subjects in Cluster 1 (61%) lived in Nuovo Leon.

### 3.3. Clusters’ Lifestyle Habits

Cluster 2 was more likely to use social media and spent much more time watching TV/surfing the internet than subjects in Cluster 1. No differences were detected in the frequency of physical activity, even though subjects in Cluster 2 were significantly more likely to declare that they tried to do physical activity more frequently after the start of the lockdown (36% in Cluster 2 vs. 24% in Cluster 1, *p*-value 0.031). Furthermore, Cluster 2 tended to have meals later than subjects in Cluster 1 (*p*-value < 0.001) and went to bed later than Cluster 1, both before and during the lockdown. Not least, an even higher proportion of subjects identified by Cluster 2 went to bed after midnight (17% of subjects in Cluster 2 went to bed after midnight before the lockdown, and the proportion became 45% during the lockdown). Finally, the prevalence of problems in falling and staying asleep during the lockdown was higher among subjects in Cluster 2 compared to those of Cluster 1 (14% in Cluster 2 vs. 4% in Cluster 1), even though nobody in Cluster 2 suffered from problems in falling and staying asleep before the lockdown. 

### 3.4. Clusters’ Dietary Habits

Cluster 1 was significantly more likely to have breakfast every day (*p*-value 0.045). Subjects identified by Cluster 2 were significantly more likely to have tacos and less likely to have fruit for dinner than subjects in Cluster 1 (15% vs. 6% had tacos, 3% vs. 33% had fruits, *p*-value < 0.001). Regarding snacking habits, Cluster 2 was likelier to have snacks than Cluster 1 (*p*-value 0.013). They most frequently had fruits/vegetables (47%) followed by bread/biscuits (14%).

### 3.5. Emotional Well-Being

Cluster 1 was significantly more likely to feel happy both before (*p*-value 0.024) and during the lockdown (*p*-value < 0.001) than subjects in Cluster 2. Subjects identified in Cluster 1 were also significantly more likely to be concentrated on improving their spiritual health (*p*-value < 0.001), and, differently from Cluster 2 subjects, they seemed to not be worried about the possibility of spending another month at home (*p*-value 0.012).

## 4. Discussion

The present work was aimed at exploring the relationship between lifestyles and the COVID-19 lockdown in a sample of people living in Mexico. This work addresses a significant gap in the existing literature by focusing on the impact of the COVID-19 lockdown on Mexican youth. This population has been little studied compared to European and North American contexts. Despite the extensive research on the effects of the lockdown, our study provides specific data on an often overlooked geographic group, identifying a cluster of subjects characterized by young age, intensive social media use, and alterations in sleep patterns. 

Consistently with other surveys conducted in Mexico and outside the country, the study involved a large proportion of female subjects of young age [23,35]. 

The sample underwent a clustering procedure that identified two well-characterized groups of survey respondents, Cluster 1 and 2. Cluster 1 included older people who reported fewer adverse effects. Cluster 2 included young subjects living in Chiapas, one of the poorest states in Mexico, who reported impaired emotional well-being and sleep patterns during the lockdown. 

Lifestyle habits and emotional well-being during the COVID-19 lockdown are widely addressed in the literature, even though data from Mexico are sparse. Several studies conducted in countries severely affected by the pandemic showed that the lockdown negatively affects dietary habits, sleep patterns, and psychological wellness, especially in the youthful population [36,37]. The findings of the present studies are consistent with national and international literature, showing that youths are more prone to suffer from the side effects of the COVID-19 lockdown [38]. 

Furthermore, the study highlights that the subjects in Cluster 2 reported problems in falling and staying asleep after the lockdown started, with a delay in both get-up time and bedtime. These findings are in line with previous studies in the field [39], showing that students are the most affected by sleep pattern disruption [40]. Several factors have been advocated to be associated with the onset of alterations in sleep patterns during the lockdown, including the imposed restrictions [41] and suffering from anxiety and depression [19]. The association with increased screen time/social media use, especially close to bedtime, has been excluded by a cross-sectional study conducted during the first lockdown in Italy [42]. However, pre-lockdown studies have shown a relationship between social media use close to bedtime and sleep disturbances [18]. Such an aspect deserves further investigation given the increased time spent on social media use and more on general digital media use described during the lockdown [43]. 

Regarding dietary habits, the cluster of subjects most severely affected by the adverse effects of the lockdown, i.e., Cluster 2, was found to eat less frequently fresh products compared to Cluster 1 and to have more frequent snacks. Such findings align with the international literature in the field, showing that lockdown is associated with lower consumption of fruits and vegetables and more frequent consumption of comfort food [10]. 

### 4.1. Study Limitations

This study presents some limitations. The most relevant ones are the small sample size and the specific socio-demographic characteristics of the sample, which limit the generalizability of study results. Although this study provides valuable insights into the impact of the COVID-19 lockdown on Mexican youth, further research with more extensive and more diverse samples is needed to confirm these findings and understand their broader applicability. Anyway, given the explorative nature of the study, no formal sample size calculation was required. Not least, the questionnaire was not validated; it underwent a pilot testing evaluation on twenty subjects. Another limitation was the use of messenger apps to share the survey; however, the coverage of messenger apps in Mexico is very good, with more than 80% of mobile phone users using WhatsApp. Finally, a follow-up survey is missing since the study was designed to be cross-sectional, but it would have been useful to understand the long-term effects of the lockdown.

### 4.2. Long-Term Implications

Although the pandemic peak is over, the results of our study remain highly relevant in the post-pandemic context for several reasons. First, the potential long-term impact on emotional well-being. The present study showed that young people experienced alterations in sleep patterns and emotional distress during isolation. These effects are likely to persist in the long term. Recent literature has recorded only small improvements in sleep alterations, stress, and anxiety after the lockdown ended [44]. Understanding these effects is essential for developing targeted public health interventions to support the community. In addition, lifestyle habits, including diet and physical activity, underwent significant changes during the pandemic. These changes may have lasting effects on physical health.

## 5. Conclusions

This study provides valuable insights into the impact of the COVID-19 lockdown on vulnerable populations in Mexico, particularly youth. The findings are limited by the small-scale nature of the survey. However, they highlight the urgent need for larger surveys in Mexico to gain a more complete understanding of these impacts, particularly concerning their long-term effects. It is critical to emphasize the importance of targeted studies of vulnerable groups to guide public health efforts and develop targeted interventions. Addressing the well-being of these populations is essential, as disruptions caused by the lockdown could have long-term consequences. These include persistent alterations in sleep patterns, emotional distress, and potential negative effects on lifestyle habits. These findings underscore the need for continued support and resources. Future research should aim to fill these gaps to better inform public health policies and interventions.

## Figures and Tables

**Table 1:** Descriptive table of the sample characteristics according to the identified Clusters.

	Cluster 1	Cluster 2	*p*-Value
	(N = 67)	(N = 88)	
Gender: female	81% (54)	84% (74)	0.57
male	19% (13)	16% (14)	
Age: <20	4% (3)	12% (11)	<0.001
21–25	21% (14)	53% (47)	
26–30	7% (5)	5% (4)	
31–40	6% (4)	9% (8)	
41–50	19% (13)	11% (10)	
51–60	30% (20)	7% (6)	
61–70	12% (8)	2% (2)	
Geographical area: Nuovo Leon	61% (41)	27% (24)	<0.001
Chiapas	9% (6)	66% (58)	
Other	30% (20)	7% (6)	
Living alone: no	94% (63)	95% (84)	0.69
yes	6% (4)	5% (4)	
Religion: SDA	84% (56)	15% (13)	<0.001
Catholic	13% (9)	69% (61)	
Christian	3% (2)	12% (11)	
Other	0% (0)	3% (3)	
**Lifestyle habits**			
Daily screen time: <2 h	45% (30)	26% (23)	0.004
2–6 h	12% (8)	34% (30)	
>6 h	34% (23)	36% (32)	
none	9% (6)	3% (3)	
Daily use of social media: yes	43% (29)	78% (69)	<0.001
no	57% (38)	22% (19)	
Keeping the phone on during the night: yes	64% (43)	82% (72)	0.013
no	36% (24)	18% (16)	
Changes in lifestyle habits: increase in physical activity frequency	24% (16)	36% (32)	0.031
improvement of dietary habits	27% (18)	16% (14)	
increase in sleep time	12% (8)	5% (4)	
other	15% (10)	8% (7)	
none	22% (15)	35% (31)	
Wake up time: before 5 A.M.	3% (2)	0% (0)	<0.001
5 A.M.–8 A.M.	82% (55)	35% (31)	
after 8 A.M.	12% (8)	62% (55)	
Breakfast time: before 8 A.M.	33% (22)	9% (8)	<0.001
8 A.M.–11 A.M.	67% (45)	90% (79)	
no breakfast	0% (0)	1% (1)	
Lunch time: before 2 P.M.	42% (28)	6% (5)	<0.001
2 P.M.–4 P.M.	58% (39)	94% (83)	
Dinner time: before 8 P.M.	60% (40)	9% (8)	<0.001
8 P.M.–10 P.M.	40% (27)	78% (69)	
after 10 P.M.	0% (0)	12% (11)	
Bedtime (before the lockdown): before 10 P.M.	52% (35)	25% (22)	<0.001
before 12 A.M.	43% (29)	58% (51)	
after 12 A.M.	3% (2)	17% (15)	
problems in falling and staying asleep	1% (1)	0% (0)	
Bedtime (lockdown): before 10 P.M.	37% (25)	2% (2)	<0.001
before 12 A.M.	46% (31)	39% (34)	
after 12 A.M.	12% (8)	45% (40)	
problems in falling and staying asleep	4% (3)	14% (12)	
Physical activity: no	42% (28)	38% (33)	0.59
yes	58% (39)	62% (55)	
Time spent doing physical activity: <30 min	45% (30)	36% (32)	0.15
>60 min	1% (1)	8% (7)	
30–60 min	54% (36)	56% (49)	
Weight gain in lockdown: yes	33% (22)	49% (43)	0.045
no	67% (45)	51% (45)	
**Dietary habits**			
Breakfast: yes	93% (62)	82% (72)	0.045
no	1% (1)	0% (0)	
sometimes	6% (4)	18% (16)	
Lunch: fast food and soft drink	3% (2)	0% (0)	0.35
soup	4% (3)	3% (3)	
salad	1% (1)	3% (3)	
salad, rice or pasta, beans, tortilla, meat, beverages	91% (61)	93% (82)	
Snack: fried snacks	4% (3)	10% (9)	0.013
bread/biscuits	13% (9)	14% (12)	
popcorn	6% (4)	7% (6)	
fruits/vegetables	36% (24)	47% (41)	
other	1% (1)	8% (7)	
none	39% (26)	15% (13)	
Dinner: cereal/milk	22% (15)	20% (18)	<0.001
meat, tacos or antojito and fresh drink	6% (4)	15% (13)	
salad	3% (2)	6% (5)	
fruits	33% (22)	3% (3)	
other	36% (24)	56% (49)	
Ready-to-eat food: sometimes	16% (11)	28% (25)	0.18
no	79% (53)	69% (61)	
yes	4% (3)	2% (2)	
Increased consumption (lockdown): cereals	9% (6)	18% (16)	0.003
fruits	43% (29)	17% (15)	
vegetables	15% (10)	26% (23)	
none	33% (22)	39% (34)	
**Emotional well-being**			
Feelings before the starting of the lockdown: happiness	36% (24)	26% (23)	0.024
restlessness	37% (25)	24% (21)	
worried	13% (9)	32% (28)	
unknown	13% (9)	18% (16)	
Feelings when the lockdown was decided: happiness	21% (14)	6% (5)	0.015
restlessness	37% (25)	43% (38)	
worried	37% (25)	50% (44)	
unknown	4% (3)	1% (1)	
Actual feelings: happiness	62% (39)	25% (21)	<0.001
restlessness	25% (16)	45% (38)	
worried	13% (8)	30% (25)	
Trying to improve the spiritual health: yes	99% (66)	77% (68)	<0.001
no	1% (1)	23% (20)	
Activities to improve spiritual health: none	1% (1)	22% (19)	<0.001
reading Bible	33% (22)	7% (6)	
praying	22% (15)	25% (22)	
keeping the faith alive	25% (17)	26% (23)	
keeping the hope alive	18% (12)	20% (18)	
Aspects of life to be improved after the end of the lockdown: family/friends relationships	10% (7)	17% (15)	<0.001
spiritual life	19% (13)	6% (5)	
lifestyle	10% (7)	15% (13)	
health	0% (0)	17% (15)	
all of them	60% (40)	45% (40)	
Feel prepared to spend another month at home: yes	82% (55)	64% (56)	0.012
no	18% (12)	36% (32)	
Anxiety while using WhatsApp: yes	96% (64)	84% (74)	0.024
no	4% (3)	16% (14)	

SDA: Seventh-day Adventist.

## Data Availability

The data presented in this study are available on request from the corresponding author.

## Supplementary Materials

The following supporting information can be downloaded at: https://www.mdpi.com/article/10.3390/bs14100906/s1, Study questionnaire; Table S1 Sample characteristics.

## References

[1] Lorenzoni G., Lanera C., Azzolina D., Berchialla P., Gregori D. (2020). Is a More Aggressive COVID-19 Case Detection Approach Mitigating the Burden on ICUs? Some Reflections from Italy. Crit. Care.

[2] Caldera-Villalobos C., Garza-Veloz I., Martínez-Avila N., Delgado-Enciso I., Ortiz-Castro Y., Cabral-Pacheco G.A., Martinez-Fierro M.L. (2020). The Coronavirus Disease (COVID-19) Challenge in Mexico: A Critical and Forced Reflection as Individuals and Society. Front. Public Health.

[3] Burki T. (2020). COVID-19 in Latin America. Lancet Infect. Dis..

[4] Almeida F. (2020). Exploring the Impact of COVID-19 on the Sustainability of Health Critical Care Systems in South America. Int. J. Health Policy Manag..

[5] Gregori D., Azzolina D., Lanera C., Prosepe I., Destro N., Lorenzoni G., Berchialla P. (2020). A First Estimation of the Impact of Public Health Actions against COVID-19 in Veneto (Italy). J. Epidemiol. Community Health.

[6] Cocuzza S., Maniaci A., Pavone P., Iannella G., Taibi R., Meccariello G., Catalano A., Vicini C. (2021). Head and Neck Cancer: The New Scenario in the Pandemic COVID-19 Period. WCRJ.

[7] Catucci A., Scognamiglio U., Rossi L. (2021). Lifestyle Changes Related to Eating Habits, Physical Activity, and Weight Status during COVID-19 Quarantine in Italy and Some European Countries. Front. Nutr..

[8] Andersen A.J., Mary-Krause M., Bustamante J.J.H., Héron M., El Aarbaoui T., Melchior M. (2021). Symptoms of Anxiety/Depression during the COVID-19 Pandemic and Associated Lockdown in the Community: Longitudinal Data from the TEMPO Cohort in France. BMC Psychiatry.

[9] González-Monroy C., Gómez-Gómez I., Olarte-Sánchez C.M., Motrico E. (2021). Eating Behaviour Changes during the COVID-19 Pandemic: A Systematic Review of Longitudinal Studies. Int. J. Environ. Res. Public Health.

[10] Bakaloudi D.R., Jeyakumar D.T., Jayawardena R., Chourdakis M. (2022). The Impact of COVID-19 Lockdown on Snacking Habits, Fast-Food and Alcohol Consumption: A Systematic Review of the Evidence. Clin. Nutr..

[11] Khan M.A., Menon P., Govender R., Samra A., Nauman J., Ostlundh L., Mustafa H., Allaham K.K., Smith J.E., Al Kaabi J.M. (2021). Systematic Review of the Effects of Pandemic Confinements on Body Weight and Their Determinants. Br. J. Nutr..

[12] Cecchetto C., Aiello M., Gentili C., Ionta S., Osimo S.A. (2021). Increased Emotional Eating during COVID-19 Associated with Lockdown, Psychological and Social Distress. Appetite.

[13] Santomauro D.F., Herrera A.M.M., Shadid J., Zheng P., Ashbaugh C., Pigott D.M., Abbafati C., Adolph C., Amlag J.O., Aravkin A.Y. (2021). Global Prevalence and Burden of Depressive and Anxiety Disorders in 204 Countries and Territories in 2020 Due to the COVID-19 Pandemic. Lancet.

[14] Stockwell S., Trott M., Tully M., Shin J., Barnett Y., Butler L., McDermott D., Schuch F., Smith L. (2021). Changes in Physical Activity and Sedentary Behaviours from before to during the COVID-19 Pandemic Lockdown: A Systematic Review. BMJ Open Sport Exerc. Med..

[15] Zaccagni L., Toselli S., Barbieri D. (2021). Physical Activity during COVID-19 Lockdown in Italy: A Systematic Review. Int. J. Environ. Res. Public Health.

[16] Wang Y., Xu J., Xie T. (2023). Social Media Overload and Anxiety among University Students during the COVID-19 Omicron Wave Lockdown: A Cross-Sectional Study in Shanghai, China, 2022. Int. J. Public Health.

[17] Lee Y., Jeon Y.J., Kang S., Shin J.I., Jung Y.-C., Jung S.J. (2022). Social Media Use and Mental Health during the COVID-19 Pandemic in Young Adults: A Meta-Analysis of 14 Cross-Sectional Studies. BMC Public Health.

[18] Fobian A.D., Avis K., Schwebel D.C. (2016). The Impact of Media Use on Adolescent Sleep Efficiency. J. Dev. Behav. Pediatr. JDBP.

[19] Alimoradi Z., Broström A., Tsang H.W., Griffiths M.D., Haghayegh S., Ohayon M.M., Lin C.-Y., Pakpour A.H. (2021). Sleep Problems during COVID-19 Pandemic and Its’ Association to Psychological Distress: A Systematic Review and Meta-Analysis. EClinicalMedicine.

[20] Pinto J., van Zeller M., Amorim P., Pimentel A., Dantas P., Eusébio E., Neves A., Pipa J., Santa Clara E., Santiago T. (2020). Sleep Quality in Times of COVID-19 Pandemic. Sleep Med..

[21] Jahrami H.A., Alhaj O.A., Humood A.M., Alenezi A.F., Fekih-Romdhane F., AlRasheed M.M., Saif Z.Q., Bragazzi N.L., Pandi-Perumal S.R., BaHammam A.S. (2022). Sleep Disturbances during the COVID-19 Pandemic: A Systematic Review, Meta-Analysis, and Meta-Regression. Sleep Med. Rev..

[22] Bruno S., Bazzani A., Marantonio S., Cruz-Sanabria F., Benedetti D., Frumento P., Turchetti G., Faraguna U. (2022). Poor Sleep Quality and Unhealthy Lifestyle during the Lockdown: An Italian Study. Sleep Med..

[23] Hernández-Nava R.G., de la Luz Sánchez-Mundo M., García-Barrientos R., Espinosa-Solis V., Villalobos-Aguayo P., Salmerón-Muñiz N.N., Anaya-Tacuba J.D. (2022). Lifestyle Changes among Mexican People during the COVID-19 Lockdown in 2020: A Cross-Sectional Study. Healthcare.

[24] Sánchez-Ortiz N.A., Colchero M.A. (2024). Changes in Food and Beverage Purchases Associated With the Coronavirus Disease Pandemic in Mexico. J. Acad. Nutr. Diet..

[25] Espinoza-Ortega A., Martínez-García C.G., Rojas-Rivas E., Fernández-Sánchez Y., Escobar-López S.Y., Sánchez-Vegas L. (2021). Consumer and Food Changes in Mexican Households during Maximal Contingency in the COVID-19 Pandemic. Int. J. Gastron. Food Sci..

[26] López-Gil J.F., Gaya A.R., Reuter C.P., Caetano C.I., Sentone R.G., Caetano H.B.S., Brazo-Sayavera J. (2021). Sleep-Related Problems and Eating Habits during COVID-19 Lockdown in a Southern Brazilian Youth Sample. Sleep Med..

[27] Aminoff S.R., Mork E., Barrett E.A., Simonsen C., ten Velden Hegelstad W., Lagerberg T.V., Melle I., Romm K.L. (2022). Locked out during COVID-19 Lockdown—An Online Survey of Relatives of People with Psychotic and Bipolar Disorders in Norway. BMC Public Health.

[28] Navarro-Pérez C.F., Fernández-Aparicio Á., González-Jiménez E., Montero-Alonso M.Á., Schmidt-RioValle J. (2022). Effects of COVID-19 Lockdown on the Dietary Habits and Lifestyle in a Population in Southern Spain: A Cross-Sectional Questionnaire. Eur. J. Clin. Nutr..

[29] Sameer A.S., Khan M.A., Nissar S., Banday M.Z. (2020). Assessment of Mental Health and Various Coping Strategies among General Population Living under Imposed COVID-Lockdown across World: A Cross-Sectional Study. Ethics Med. Public Health.

[30] Instituto Federal de Telecomunicaciones (Mexico), Primera Encuesta 2020, Usuarios de Servicios de Telecomunicaciones. 2020.

[31] Di Franco G. (2016). Multiple Correspondence Analysis: One Only or Several Techniques?. Qual. Quant..

[32] Kassambara A. *Practical Guide to Cluster Analysis in R: Unsupervised Machine Learning*; Sthda: 2017; Volume 1.

[33] Lê S., Josse J. (2008). FactoMineR: An R Package for Multivariate Analysis. J. Stat. Softw..

[34] Kassambara A. *Factoextra: Visualization of the Outputs of a Multivariate Analysis*, R Package Version 1.0.; New York, NY, USA, 2015.

[35] Di Renzo L., Gualtieri P., Pivari F., Soldati L., Attinà A., Cinelli G., Leggeri C., Caparello G., Barrea L., Scerbo F. (2020). Eating Habits and Lifestyle Changes during COVID-19 Lockdown: An Italian Survey. J. Transl. Med..

[36] Wang C., Pan R., Wan X., Tan Y., Xu L., Ho C.S., Ho R.C. (2020). Immediate Psychological Responses and Associated Factors during the Initial Stage of the 2019 Coronavirus Disease (COVID-19) Epidemic among the General Population in China. Int. J. Environ. Res. Public Health.

[37] Di Renzo L., Gualtieri P., Cinelli G., Bigioni G., Soldati L., Attinà A., Bianco F.F., Caparello G., Camodeca V., Carrano E. (2020). Psychological Aspects and Eating Habits during COVID-19 Home Confinement: Results of EHLC-COVID-19 Italian Online Survey. Nutrients.

[38] Ettman C.K., Abdalla S.M., Cohen G.H., Sampson L., Vivier P.M., Galea S. (2020). Prevalence of Depression Symptoms in US Adults Before and during the COVID-19 Pandemic. JAMA Netw. Open.

[39] Alfonsi V., Gorgoni M., Scarpelli S., Zivi P., Sdoia S., Mari E., Quaglieri A., Ferlazzo F., Giannini A.M., De Gennaro L. (2021). Changes in Sleep Pattern and Dream Activity across and after the COVID-19 Lockdown in Italy: A Longitudinal Observational Study. J. Sleep Res..

[40] Marelli S., Castelnuovo A., Somma A., Castronovo V., Mombelli S., Bottoni D., Leitner C., Fossati A., Ferini-Strambi L. (2021). Impact of COVID-19 Lockdown on Sleep Quality in University Students and Administration Staff. J. Neurol..

[41] Altena E., Baglioni C., Espie C.A., Ellis J., Gavriloff D., Holzinger B., Schlarb A., Frase L., Jernelöv S., Riemann D. (2020). Dealing with Sleep Problems during Home Confinement Due to the COVID-19 Outbreak: Practical Recommendations from a Task Force of the European CBT-I Academy. J. Sleep Res..

[42] Cellini N., Canale N., Mioni G., Costa S. (2020). Changes in Sleep Pattern, Sense of Time and Digital Media Use during COVID-19 Lockdown in Italy. J. Sleep Res..

[43] Ting D.S.W., Carin L., Dzau V., Wong T.Y. (2020). Digital Technology and COVID-19. Nat. Med..

[44] Salfi F., Amicucci G., Corigliano D., Viselli L., D’Atri A., Tempesta D., Gorgoni M., Scarpelli S., Alfonsi V., Ferrara M. (2023). Two Years after Lockdown: Longitudinal Trajectories of Sleep Disturbances and Mental Health over the COVID-19 Pandemic, and the Effects of Age, Gender and Chronotype. J. Sleep Res..

