# Supplementary material for: Sleep Patterns, Social Media Usage, and Dietary Habits during COVID-19 Lockdown in Mexico: A Cross-Sectional Study
Source: Behav Sci (Basel). 2024 Oct 8;14(10):906. doi: 10.3390/bs14100906 (PMC11504667; doi:10.3390/bs14100906)
Supplement: Supplementary file 1 [file behavsci-14-00906-s001.zip › behavsci-3168276-supplementary.pdf]

Supplementary Material

Estilo de vida en familias mexicanas durante COVID-19

EV-FAMMEX-COVID-19

Gender

- Female
- Male

Age group (years)

- <20
- 21-25
- 26-30
- 31-40
- 41-50
- 51-60
- 61-70
- >70

Religion

- Catholic
- Adventist
- Christian
- Atheist
- Other

Where do you live?

- Aguascalientes
- Baja California norte
- Baja California sur
- Campeche
- Chiapas
- Chihuahua
- Coahuila de Zaragoza
- Colima
- Durango
- Estado de México
- Guanajuato
- Guerrero
- Hidalgo

- Jalisco
- Michoacán
- Morelos
- Nayarit
- Nuevo León
- Oaxaca
- Puebla
- Querétaro
- Quintana Roo
- San Luis Potosí
- Sinaloa
- Sonora
- Tabasco
- Tamaulipas
- Tlaxcala
- Veracruz
- Yucatán
- Zacatecas

Do you live alone?

- No
- Yes

Of the following diseases, which one(s) do you suffer from?

- Overweight
- Obesity
- Diabetes
- Hypertension
- Gastritis
- Gastric ulcers
- Colitis
- Cancer
- Hepatic steatosis

- Other
- None

How did you feel before the lockdown?

- Worried
- Happy
- Hopeful
- Hopeless
- Feelings of uncertainty
- Restless
- I can’t express how I felt

How did you feel when the lockdown started?

- Worried
- Happy
- Hopeful
- Hopeless
- Feelings of uncertainty
- Restless
- I can’t express how I felt

What do you feel about the news delivered by social media?

- Worry
- Alertness
- Emotional instability
- Willingness to spread the information through my social networks
- Indifference
- Unbelief
- Other

How do you feel right now?

- Worried
- Happy
- Hopeful
- Hopeless
- Feelings of uncertainty
- Restless
- Tired
- I can’t express how I feel

How do your family and friends help you?

- Sending messages of hope through social networks, telephone, the internet
- Keeping in touch every day
- Praying for me
- Talking to each other and sending messages of peace and hope
- Talking to each other and sending messages of fear and anxiety

How do you support your family and friends?

- Sending messages of hope through social networks, telephone, the internet
- Keeping in touch every day
- Praying for them
- Sending peace and hope
- Let everyone take care of themselves as they can

How much water do you drink per day?

- < 2 glasses
- 2-5 glasses
- 6-8 glasses
- >8 glasses

At what time did you go to bed before the lockdown?

- Before 10 pm

- Before 12 am
- After 12 am
- Problems in falling and staying asleep

At what time do you go to bed?

- Before 10 pm
- Before 12 am
- After 12 am
- Problems in falling and staying asleep

How many hours do you watch TV/Internet per day?

- < 2 hours
- 2-6 hours
- >6 hours
- None

Do you use social networks daily?

- Yes
- No

Do you feel worried/anxious when you receive a Whatsapp message?

- Yes
- No

Do you immediately share the message received?

- Yes
- No

Do you constantly look at your cell phone and wait for messages?

- Yes
- No

Do you keep your cell phone on all night?

- Yes
- No

At what time do you get up in the morning?

- Before 5 am
- Between 5 - 8 am
- After 8 am

Do you exercise every day?

- Yes
- No

How much time do you spend doing physical activity?

- Less than 30 minutes
- 30-60 minutes
- More than 60 minutes

Please specify the type of physical activity:

Do you exercise with your cohabitants?

- Yes
- No
- Sometimes

Do you think it is important to exercise during the lockdown?

- Yes
- No

Do you have breakfast every day?

- Yes
- No

- Sometimes

What do you have for breakfast?

- Coffee
- Fruits
- Cereals, fruits, and milk
- Eggs, cereals, fruits, and bread
- Variety of foods
- Other

At what time do you have breakfast?

- Before 8 am
- 8 - 11 am
- I don't have breakfast

At what time do you have lunch?

- Before 2 pm
- 2 - 4 pm

What do you have for lunch?

- Salad
- Soup
- Salad, rice or pasta, meat, beans, tortilla, beverages
- Salad and soup
- Fast food and soft drink

At what time do you have dinner?

- Before 8 pm
- 8 -10 pm
- After 10 pm

What do you have for dinner?

- Cereals with milk
- Meat, tacos or antojitos, and fresh drinks
- Salad
- Fruits
- Other

What do you have for a snack?

- Fried snacks
- Bread and biscuits
- Popcorn
- Fruits and vegetables
- Other
- None

Do you eat ready-to-eat food every day?

- Yes
- No
- Sometimes

Is there a difference in food intake compared to before the lockdown?

- Yes
- No
- Sometimes

Do you gain weight?

- Yes
- No

Do you increase the budget for food during the lockdown?

- Yes

- No
- Sometimes

Which foods do you include in your daily diet during the lockdown that you did not eat before?

- Cereals
- Fruits
- Vegetables
- None

Is there a change in one of the following habits?

- Increase in physical activity frequency
- Improvement of dietary habits
- Increase in sleep time
- Weight control
- Other
- None

What are the effects of the lockdown on your family relationships?

- The lockdown allows for much closer family relationships
- The lockdown allows for more extended time spent at home with the partner
- The lockdown allows for reflections on the importance of the family
- The lockdown allows for reflections on what to do when it is over

Does the lockdown allow you to think much more about your spiritual health?

- Yes
- No

What are you doing to improve your spiritual health?

- Nothing
- Reading the Bible
- Praying

- Keeping the faith alive
- Keeping the hope alive

What aspects of your life do you want to improve when the lockdown is over?

- Family and Friends relationships
- Spiritual life
- Lifestyle
- Health
- All of them

How do you feel right now?

- Calm and confident
- Hopeful
- Hopeless
- Alone
- Need help

What aspects not considered in this questionnaire do you think are relevant to be addressed during the lockdown?

____

What do you do during the lockdown?

- Reading
- Studying
- Planting and growing vegetables
- Houseworks
- Working
- Other

Do you feel prepared to spend another month at home?

- Yes

- No

Table S1. Sample characteristics

	Overall
	(N=155)
Gender: *female*	83% (128)
male	17% (27)
Age: *<20*	9% (14)
21-25	39% (61)
26-30	6% (9)
31-40	8% (12)
41-50	15% (23)
51-60	17% (26)
61-70	6% (10)
Living alone: *no*	95% (147)
yes	5% (8)
Religion: *ASD*	45% (69)
Catholic	45% (70)
Christian	8% (13)
Other	2% (3)
Lifestyle habits	
Daily screen time: *<2 hours*	34% (53)
2-6 hours	25% (38)
>6 hours	35% (55)
none	6% (9)
Daily use of social media: *yes*	63% (98)
no	37% (57)
Keeping the phone on during the night: *yes*	74% (115)
no	26% (40)
Changes of lifestyle habits: *increase of physical activity frequency*	31% (48)
improvement of dietary habits	21% (32)
increase of time spent sleeping	8% (12)
other	11% (17)

none	30% (46)
Wake up time: *before 5 AM*	1% (2)
5 AM - 8 AM	55% (86)
after 8 AM	41% (63)
Breakfast time: *before 8 AM*	19% (30)
8 AM - 11 AM	80% (124)
no breakfast	1% (1)
Lunch time: *before 2 PM*	21% (33)
2 PM - 4 PM	79% (122)
Dinner time: *before 8 PM*	31% (48)
8 PM - 10 PM	62% (96)
after 10 PM	7% (11)
Bedtime (before the lockdown): *before 10 PM*	37% (57)
before 12 AM	52% (80)
after 12 AM	11% (17)
sleep disturbances	1% (1)
Bedtime (lockdown): *before 10 PM*	17% (27)
before 12 AM	42% (65)
after 12 AM	31% (48)
sleep disturbances	10% (15)
Physical activity: *no*	39% (61)
yes	61% (94)
Time spent doing physical activity: < *30min*	40% (62)
> *60min*	5% (8)
30-60 min	55% (85)
Weight gain in lockdown: *yes*	42% (65)
no	58% (90)
Dietary habits	
Breakfast: y*es*	86% (134)
no	1% (1)
sometimes	13% (20)
Lunch: *take away and soft drink*	1% (2)
soup	4% (6)

salad	3% (4)
salad, rice or pasta, beans, tortilla, meat	92% (143)
Snack: *fried snacks*	8% (12)
bread/biscuits	14% (21)
popcorn	6% (10)
fruits/vegetables	42% (65)
other	5% (8)
none	25% (39)
Dinner: *cereal/milk*	21% (33)
meat, tacos or antojito and fresh drink	11% (17)
salad	5% (7)
fruits	16% (25)
other	47% (73)
Ready-to-eat food: *sometimes*	23% (36)
no	74% (114)
yes	3% (5)
Increase of food budget (lockdown): *whole grains*	14% (22)
fruits	28% (44)
vegetables	21% (33)
no	36% (56)
Emotional wellbeing	
Feelings before the starting of the lockdown: *happiness*	30% (47)
restlessness	30% (46)
worried	24% (37)
unknown	16% (25)
Feelings when the lockdown was decided: *happiness*	12% (19)
restlessness	41% (63)
worried	45% (69)
unknown	3% (4)
Actual feelings: *happiness*	41% (60)
restlessness	37% (54)
worried	22% (33)
Trying to improve the spiritual health: *yes*	86% (134)

no	14% (21)
Activities to improve spiritual health: *none*	13% (20)
reading Bible	18% (28)
praying	24% (37)
keeping the faith alive	26% (40)
keeping the hope alive	19% (30)
Aspects of life to be improved after the end of the lockdown: *family/friends relationship*s	14% (22)
spiritual life	12% (18)
lifestyle	13% (20)
health	10% (15)
all of them	52% (80)
Feel prepared to spend another month at home: *yes*	72% (111)
no	28% (44)
Anxiety while using WhatsApp: yes	89% (138)
no	11% (17)
